# Lifestyle intervention during pregnancy in patients with gestational diabetes mellitus and the risk of neonatal hypoglycemia: A systematic review and meta-analysis

**DOI:** 10.3389/fnut.2022.962151

**Published:** 2022-07-28

**Authors:** Ya-Hai Wang, Huan-Huan Zhou, Zhibin Nie, Jingwang Tan, Zicheng Yang, Shengliang Zou, Zheng Zhang, Yu Zou

**Affiliations:** ^1^School of Arts and Physical Education, Nanchang Normal College of Applied Technology, Nanchang, Jiangxi, China; ^2^Hubei Key Laboratory of Food Nutrition and Safety, Department of Nutrition and Food Hygiene, Tongji Medical College, Huazhong University of Science and Technology, Wuhan, China; ^3^Department of Nutrition and Food Hygiene and MOE Key Lab of Environment and Health, School of Public Health, Tongji Medical College, Huazhong University of Science and Technology, Wuhan, China; ^4^Department of Sport and Exercise Science, College of Education, Zhejiang University, Hangzhou, China; ^5^Center of Child Health Management, Children's Hospital of Soochow University, Suzhou, China

**Keywords:** lifestyle intervention, gestational diabetes mellitus, neonatal hypoglycemia, systematic review, meta-analysis

## Abstract

**Objective:**

Neonatal hypoglycemia is a severe adverse consequence of infants born to mothers with gestational diabetes mellitus (GDM), which can lead to neonatal mortality, permanent neurological consequences, and epilepsy. This systematic review and meta-analysis of randomized controlled trials (RCTs) was conducted to explore the effect of lifestyle intervention during pregnancy in women with GDM on the risk of neonatal hypoglycemia.

**Methods:**

PubMed, Web of Science, Cochrane Library, CINAHL, and SPORTDiscus databases were searched by 1st April 2022. Data were pooled as the risk ratio (RR) with 95% CIs of neonatal hypoglycemia. Random-effects, subgroup analyses, meta-regression analysis, and leave-one-out analysis were conducted, involving 18 RCTs.

**Results:**

Prenatal lifestyle intervention could significantly reduce the risk of neonatal hypoglycemia (RR: 0.73, 95% CI: 0.54–0.98, *P* = 0.037). Subgroup analysis further demonstrated that the reduced risk of neonatal hypoglycemia was observed only when subjects were younger than 30 years, initiated before the third trimester, and with dietary intervention. Meta-regression analysis revealed that the risk of neonatal hypoglycemia post lifestyle intervention was lower in mothers with lower fasting glucose levels at trial entry.

**Conclusion:**

We found that prenatal lifestyle intervention in women with GDM significantly reduced the risk of neonatal hypoglycemia. Only lifestyle intervention before the third trimester of pregnancy, or dietary intervention only could effectively reduce the risk of neonatal hypoglycemia. Future studies are required to explore the best pattern of lifestyle intervention and to determine the proper diagnostic criteria of GDM in the first/second trimester of pregnancy.

**Systematic review registration:**

https://www.crd.york.ac.uk/PROSPERO/#myprospero, PROSPERO, identifier: CRD42021272985.

## Introduction

Gestational diabetes mellitus (GDM), defined as hyperglycemia with onset or first recognized during pregnancy, is associated with an increased risk of a range of adverse outcomes for offspring that can be passed on from generation to generation (1), namely, hypoglycemia (2), obesity, and type 2 diabetes (3–5). The incidence rate of GDM ranged from 3.4% to 37.7%, depending on different diagnostic criteria and population (6). A population-based cohort study showed that infants born to mothers with GDM had a significantly increased risk of hypoglycemia (OR: 11.71, 95% CI: 7.49–18.30) (7). The high concentration of blood glucose in the mother leads to an increase in fetal glucose intake, which stimulates excess fetal insulin secretion, thereby inducing neonatal hypoglycemia (8). Neonatal hypoglycemia is an important factor in neonatal mortality (9) and permanent neurological consequences (9, 10). Even infants who are slightly and transiently exposed to hypoglycemia are at risk of later delayed neurodevelopment (11–15). Severe neonatal hypoglycemia can also lead to epilepsy, personality disorder, impaired heart function, and muscle weakness (9).

Lifestyle interventions, mainly dietary interventions, also including physical exercise interventions and other interventions, are usually the first-line strategy for managing GDM (16). A systematic review and meta-analysis of 19 controlled trials showed that dietary intervention during pregnancy effectively reduced the incidence rate of GDM (17). After being diagnosed with GDM, 70%−85% of patients were efficient to control blood glucose by lifestyle intervention *per se* according to American Diabetes Association (18). Studies have shown that the adverse perinatal outcomes caused by GDM might be improved with the treatment of GDM (19). Despite that, the effect of prenatal lifestyle intervention on the incidence of neonatal hypoglycemia remains inconclusive, although mounting studies have sprung up. Some results from human studies of randomized controlled trials (RCTs) suggested that lifestyle intervention could effectively reduce the risk of neonatal hypoglycemia (20–22). Recently, a large well-conducted RCTs revealed that the overall neonatal complications were significantly reduced by 47% post-smartphone-based lifestyle intervention (23). While some other evidences from human RCTs reported null effects on the risk of neonatal hypoglycemia (24–26).

Thus, the purpose of this review was to evaluate the effect of lifestyle intervention during pregnancy in women with GDM on the hypoglycemia risk of their neonate and to examine related influencing factors.

## Methods

### Literature search

This meta-analysis was carried out in compliance with the Preferred Reporting Items for Systematic Reviews and Meta-Analysis (PRISMA 2020) guidelines (27). The protocol number of this study is PROSPERO CRD 42021272985. Relevant articles published through 1st April 2022 were searched from PubMed, Web of Science, Cochrane Library, Cumulated Index to Nursing and Allied Health Literature (CINAHL), and SPORTDiscus (a full-text database of sports and sports medicine journals in the EBSCOhost), based on the Population, Intervention, Comparator, Outcome and Study design (PICOS) framework. The following search strategy was used: (lifestyle OR diet OR “physical exercise”) AND (pregnancy OR “diabetes mellitus”) AND “neonatal hypoglycemia” AND “randomized controlled trial.” Detailed search terms are given in Supplementary Table 1.

### Study selection

Two authors (YHW and HHZ) independently screened titles and abstracts, then reviewed the full text of all relevant studies for eligibility. The third researcher (YZ OR ZZ) arbitrated any discrepancies to reach a consensus. We included RCTs that evaluated the effect of lifestyle intervention during pregnancy on patients diagnosed with GDM with reported neonatal hypoglycemia. Inclusion criteria for considering studies for this review were: (1) types of studies: published randomized controlled trials; (2) types of participants: pregnant women diagnosed with GDM (defined by trialists); (3) types of interventions: lifestyle interventions (dietary intervention with or without those following interventions: exercise intervention, health education, self-monitoring of blood glucose, etc.) vs. placebo or usual care; and (4) types of outcome measures: neonatal hypoglycemia. The exclusion criteria included: (1) types of studies: quasirandomized trials or animal studies or reviews; (2) types of participants: patients with type 1 or type 2 diabetes before pregnancy, healthy subjects; (3) types of intervention: comparing different lifestyle interventions; and (4) types of outcome measures: lacking the results of neonatal hypoglycemia or sufficient data to calculate the results of neonatal hypoglycemia. We also conducted a manual search for reference lists of the included articles.

### Data extraction and quality assessment

Data extraction and quality assessment were conducted by two trained people (YHW and HHZ) independently. The data were extracted to a form we designed, including: the first author's surname, publication year, study design, study location, sample size (intervention/comparators), age at pregnancy, gestational age at baseline, mother's fasting glucose level at baseline, maternal body mass index (BMI) (kg/m^2^) at baseline or prepregnancy, intervention information, comparator information, and the outcome of neonatal hypoglycemia. Studies containing two or more intervention strata were analyzed as separate trials. The risk of bias (RoB) of each included study was assessed according to the criteria of the Cochrane Handbook for Systematic Reviews of Interventions (28). The Grading of Recommendation, Assessment, Development, and Evaluation (GRADE) approach was used to assess the quality of evidence (29). According to the GRADE handbook, study design dictates the baseline quality of the evidence (RCTs are initially assigned a ranking of high), and other factors could downgrade (risk of bias, inconsistency, indirectness of evidence, imprecision, and publication bias) or upgrade (large effect size, plausible residual confounding, and dose-response relationship) the quality of evidence. Discrepancies were resolved through discussion or by involving the third reviewer (YZ, OR, and ZZ).

### Statistical analysis

A fixed-effect meta-analysis was used to pool estimates of summary risk ratio (RR) with 95% CIs of neonatal hypoglycemia. If substantial statistical heterogeneity was detected, the random-effects model was used to summarize the overall effect. Advanced data extraction was performed for studies that did not directly provide the mean and SD of continuous variables (30). The heterogeneity among studies was tested using the chi^2^ test and quantified by *I*^2^-statistic (31). The presence of heterogeneity was indicated by *P*-value < 0.10 in the chi^2^ test or *I*^2^ > 30%.

Sources of potential heterogeneity were investigated by subgroup analyses and meta-regression based on age at pregnancy, gestational age at baseline, maternal fasting glucose level at baseline, and intervention types. The *P*-value < 0.1 was considered statistically significant in meta-regression analysis. Sensitivity analyses based on leave-one-out cross-validation were conducted to assess the robustness of the results in primary meta-analyses and to evaluate the impact of each trial on the heterogeneity, using a *p*-value < 0.05 as the criterion (32). Begg's and Egger's regression tests and funnel plots were used to assess possible publication bias. The *P*-value < 0.10 suggested the presence of publication bias (33). If publication bias was found, the trim and fill method was utilized (34).

Data analyses were performed using STATA version 11.0 (Stata Corp, College Station, TX, USA), with double data input to avoid input errors. The risk of bias in included studies was assessed using RevMan version 5.3 (Cochrane Collaboration, Oxford, UK). *P* < 0.05 was deemed as statistically significant unless specified elsewhere.

## Results

### Literature search and study characteristics

**Figure 1:**
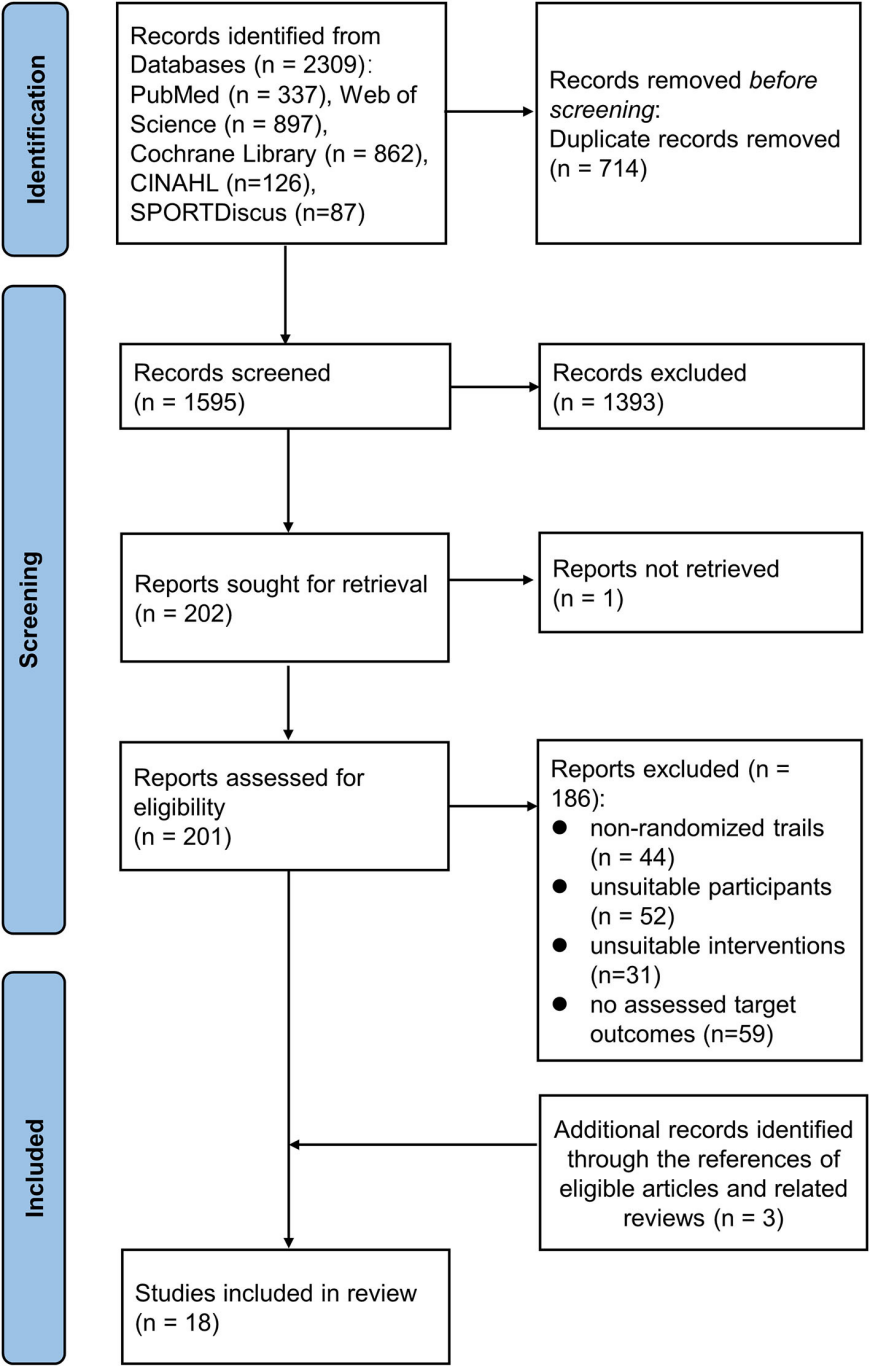
Flowchart of study selection process through the review.

As shown in Figure 1, the detailed process of literature search and study selection is presented in the flowchart. A total of 2,309 articles (337 from PubMed, 897 from Web of Science, 862 from Cochrane Library, 126 from CINAHL, and 87 from SPORTDiscus) were identified through initial searching, out of which 714 studies were removed because of duplication. Then 1,393 records were excluded after screening the title/abstract. The remaining 186 articles were eliminated for the following reasons: 44 studies were excluded due to inappropriate article design, 52 studies did not meet the inclusion criteria due to unsuitable participants, 31 studies lacked proper treatment, and 59 studies lacked sufficient data. Additional 3 studies were included through evaluating the reference lists of the included articles. Eventually, 18 eligible studies were included in the final quantitative synthesis.

### Characteristics of included studies

Characteristics of 18 studies (19–26, 35–44) included in this meta-analysis are shown in Table 1, involving a total of 5,182 women and 4,945 newborns. Sample sizes ranged from 45 (35) to 1,030 (19) newborns. 13 studies (20, 21, 24, 25, 35–42, 44) had a sample size of fewer than 300 newborns. Five studies were conducted in China (21, 22, 24, 36, 43), four in Iran (25, 35, 38, 44), two each in the United States (26, 42) and Australia (19, 41), and one each in the United Arab Emirates (20), Canada (37), Egypt (39), the United Kingdom (40), and Singapore (23). All studies reported data for maternal age. The mean maternal age of the intervention group ranged from 26.88 ± 3.15 (21) to 31.70 ± 4.00 (23) years. In the control group, the mean maternal age ranged from 26.20 ± 3.10 (44) to 32.20 ± 4.40 (23) years. Studies were initiated in the first (*n* = 3) (36, 39, 43), second (*n* = 6) (21, 23, 25, 35, 38, 40), and third (*n* = 6) (19, 22, 24, 26, 41, 42) trimesters, while three studies (20, 37, 44) were unspecified. Specific gestational age at trial entry was reported in 13 studies (19, 21–24, 26, 35, 36, 38, 40–43), with a mean age ranging from 10.00 ± 0.32 (36) to 30.46 ± 4.71 (24) weeks. The specific fasting glucose level at trial entry was reported in 14 studies (19, 21–26, 36, 37, 39–43), with a mean level ranging from 4.65 ± 0.50 (23) to 10.05 ± 1.61 (37) mmol/L. Maternal BMI at trial entry and before pregnancy was reported in eight studies (19, 24, 25, 38, 39, 41, 42, 44) and seven studies (22, 23, 26, 35, 36, 40, 43), respectively. As for treatments of the intervention groups, the comprehensive intervention was utilized in seven studies (19–21, 23, 24, 26, 43), a calorie/carbohydrate-restricted diet was utilized in three studies (37, 41, 42), vitamin D supplements were utilized in two studies (25, 35), probiotics were utilized in two studies (25, 44), and phytosterol-enriched food (36), omega-3 fatty acids plus vitamin E supplements (38), vitamin C supplements (39), docosahexaenoic acid (DHA) supplements (40), and epigallocatechin 3-gallate (EGCG) supplements (22) were used in every single study.

**Table 1:** Characteristics of included studies in this meta-analysis (18 studies).

**Study**	**Design**	**Country**	**Sample (intervention/** ** comparators)**	**Age at pregnancy**	**Gestational age at baseline (weeks)**	**Fasting glucose level at baseline (mmol/L)**	**Interventions**	**Comparators**
Asemi et al. (35)	RP, Db	Iran	Mothers: 22/23 Newborns: 22/23	30.95	25.56	>5.23	VD supplements (50,000 IU VD3 pearl 2 times: at study baseline and day 21 of intervention)	Placebo (2 placebos at the mentioned times)
Cao et al. (24)	RP	China	Mothers: 127/148 Newborns: 127/148	30.39	30.46	4.79	Comprehensive intensive therapy (individualized diabetes education, lifestyle intervention, scheduled clinic visits, strict glucose control, and frequent glucose self-monitoring)	Standard therapeutic regimen (group education and instruction the importance of proper diet, exercise, and self-monitoring of glucose level)
Crowther et al. (19)	RP, Db	Australia	Mothers: 490/510 Newborns: 506/524	30.49	29.12	4.80	Individualized dietary advice, blood glucose monitoring +/- insulin therapy	Usual care
Elnour et al. (20)	RP	United Arab Emirates	Mothers: 99/66 Newborns: 99/66	30.94	8–19	/	Structured pharmaceutical care, structured education on diet, exercise and diabetes treatment, self-monitoring of blood glucose	Traditional services (monthly clinic visits and self-monitoring of plasma glucose)
Gao et al. (36)	RP, Db	China	Mothers: 123/121 Newborns: 123/121	30.64	10.00	5.71	Phytosterol-enriched spreads, 20g/day, contains 4 g of phytosterols/day	Regular margarine spread, 20g/day
Garner et al. (37)	DP, Sb	Canada	Mothers: 149/150 Newborns: 149/150	30.70	24–32	10.05	Calorie–restricted diet of 35 kcal/kg ideal body weight per day	Unrestricted healthy diet
Jamilian et al. (25)	RP, Db	Iran	Mothers: 30/28 Newborns: 30/28	29.38	24–28	5.27	VD (50,000 IU/every 2weeks) + probiotic (8 × 109 CFU/day)	Placebo
Jamilian et al. (25)	RP, Db	Iran	Mothers: 29/28 Newborns: 29/28	30.56	24–28	5.30	probiotic (8 × 109 CFU/day)	Placebo
Jamilian et al. (38)	RP, Db	Iran	Mothers: 30/30 Newborns: 29/30	30.05	26.10	>5.11	1,000 mg omega-3 fatty acids from flaxseed oil + 400 IU VE supplements	Placebo
Karamali et al. (38)	RP, Db	Iran	Mothers: 30/30 Newborns: 30/30	26.70	/	/	Synbiotic capsule containing Lactobacillus acidophilus strain T16 (IBRC-M10785), L. casei strain T2 (IBRC-M10783), and Bifidobacterium bifidum strain T1 (IBRC-M10771) (2 × 109CFU/g each) + 800 mg inulin (HPX)	Placebo
Landon et al. (26)	RP	USA	Mothers: 485/473 Newborns: 381/357	29.05	28.85	4.80	Nutritional counseling and diet therapy +/- insulin plus self-monitoring of blood glucose	Usual care +/- insulin plus self-monitoring of blood glucose
Maged et al. (39)	RP, Sb	Egypt	Mothers: 100/100 Newborns: 100/100	27.40	10–12	5.06	1 g L-ascorbic acid/day	Placebo
Meng et al. (21)	RP	China	Mothers: 45/48 Newborns: 45/48	26.88	24.12	5.04	Comprehensive nursing intervention (psychological intervention, health education, diet control, exercise intervention, pregnancy monitoring, and prevention of postpartum complications)	Routine nursing (one-off health education and nutrition and exercise guidance, regular pregnancy monitoring, regular postpartum care)
Min et al. (40)	RP, Db	UK	Mothers: 67/71 Newborns: 58/56	32.25	26.85	5.55	2 capsules of DHA-enriched formula/day	2 capsules of high oleic acid sunflower seed oil/day
Rae et al. (41)	RP, Db	Australia	Mothers: 66/58 Newborns: 59/50	30.39	28.19	4.85	Moderately energy restricted diabetic diet providing between 6,800 and 7,600 kJ/day	Diabetic diet which was not energy restricted, providing approximately 8,600–9,500 kJ/day
Trout et al. (42)	RP	USA	Mothers: 37/31 Newborns: 37/31	28.88	29.78	5.07	Lower-carbohydrate diet (35–40% of total calories)	Usual pregnancy diet (50–55% carbohydrate)
Yang et al. (43)	RP, Db	China	Mothers: 339/361 Newborns: 339/361	29.80	10.80	5.05	Shared care (Individualized dietary and physical activity counseling, energy intakes recommendation, moderate physical activity daily, self-monitoring blood glucose +/-insulin)	Usual care (hospital-based education session +/- insulin)
Yew et al. (23)	RP, Sb	Singapore	Mothers: 170/170 Newborns: 168/165	31.95	26.85	4.65	Usual care + Habits-GDM app (integrated dietary, physical activity, weight, and glucose monitoring)	Usual care (hospital-based education session, self-monitoring of blood glucose +/- insulin)
Zhang et al. (22)	RP, Db	China	Mothers: 176/150 Newborns: 176/175	29.19	29.00	5.81	500 mg of EGCG/day	Placebo

*Db, double blind; DHA, docosahexaenoic acid; EGCG, epigallocatechin 3-gallate; RC, randomized crossover; RP, randomized-parallel; Sb, single blind; UK, United Kingdom; USA, United States of America, VC, vitamin C; VD, vitamin D; VE, vitamin E*.

### Excluded studies

In total, six studies (45–50) included subjects without GDM, additional two studies (51, 52) included women with impaired glucose tolerance but did not meet the diagnosis of GDM as defined by trialists. Two studies (53, 54) did not use an intervention/comparison included in this review.

### Risk of bias in included studies

**Figure 2:**
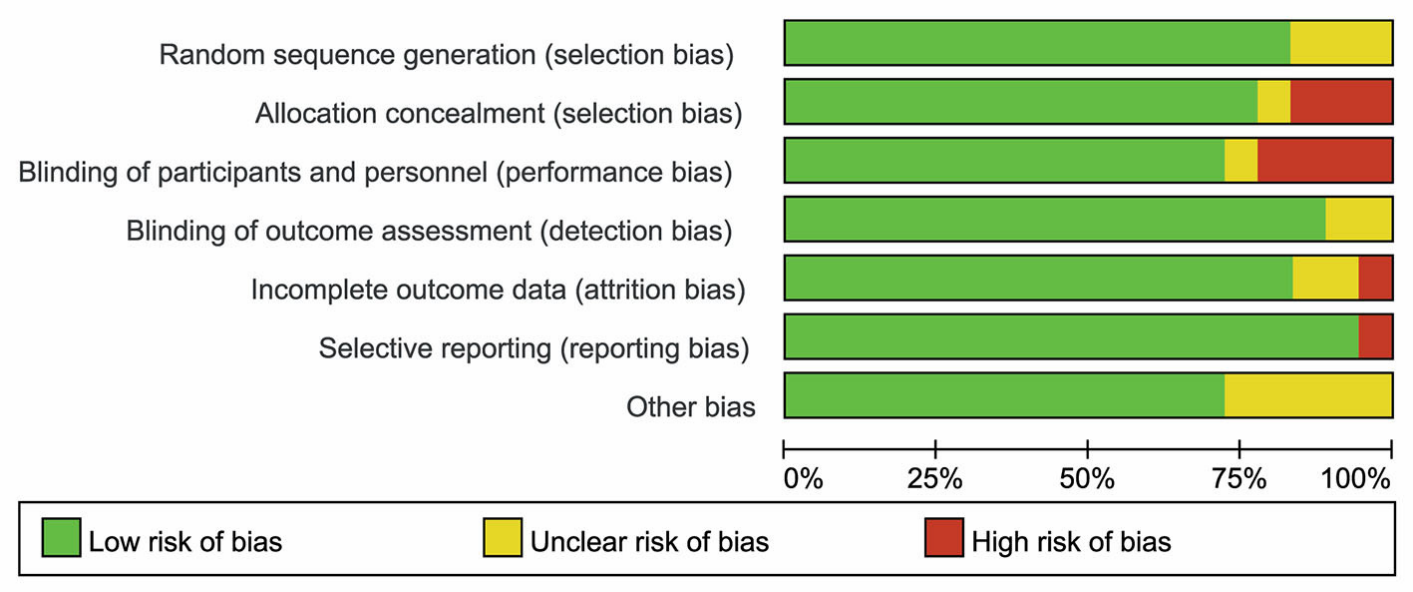
Risk of bias of summary was assessed using the risk of bias (RoB) tool of the Cochrane Handbook for Systematic Reviews of Interventions.

**Figure 3:**
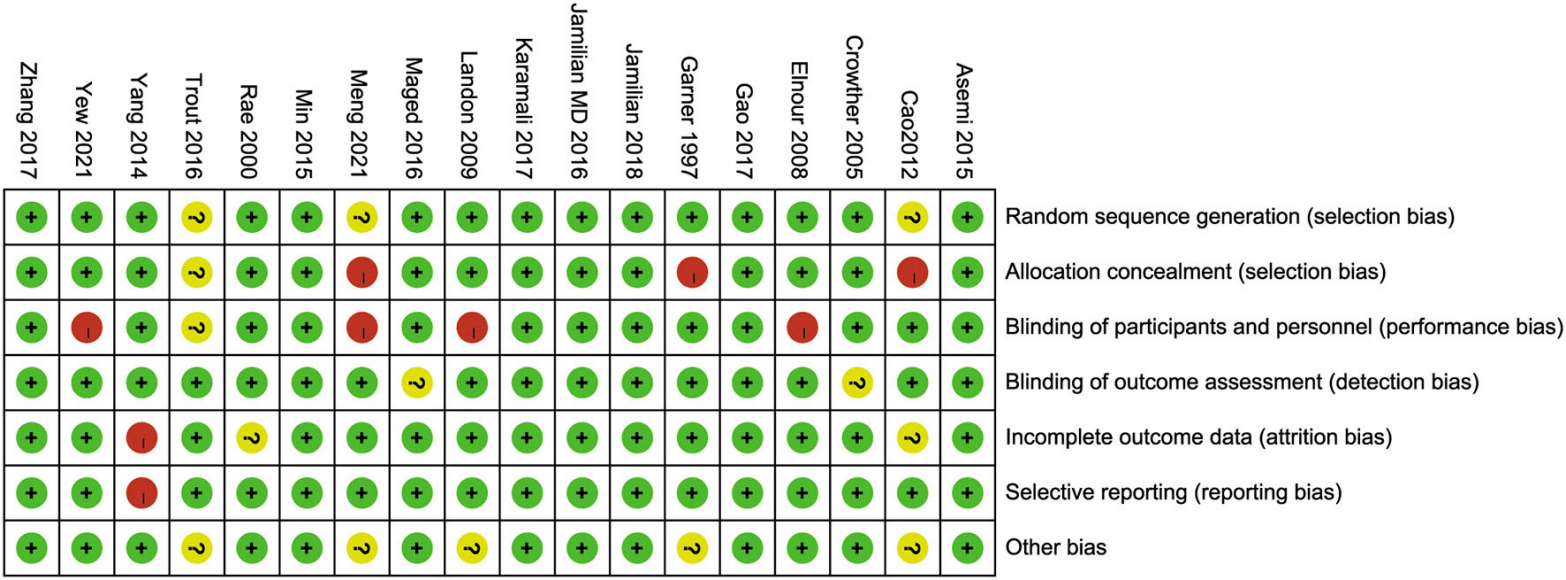
Risk of bias of each included study was assessed using the risk of bias (RoB) tool of the Cochrane Handbook for Systematic Reviews of Interventions.

As shown in Figures 2, 3, three studies (21, 24, 42) were considered to be of unclear risk of bias for randomization, the other 15 (19, 20, 22, 23, 25, 26, 35–41, 43, 44) studies were at low risk of bias. Three studies (21, 24, 37) were considered to be of high risk of bias for allocation concealment, one study (42) was judged to be of unclear risk of bias. Four studies (20, 21, 23, 26) had a high risk of performance bias, one study (42) was judged to be of unclear risk of bias. Two studies (19, 39) had an unclear risk of detection bias. One study (43) was judged as high risk for attrition bias and two studies (24, 41) were considered to be of unclear risk for attrition bias. One study (43) was considered to be of high risk for reporting bias. Five studies (21, 24, 26, 37, 42) had unclear risk of other biases (potential biases related to the study design).

### Effect of lifestyle intervention on the risk of neonatal hypoglycemia

**Figure 4:**
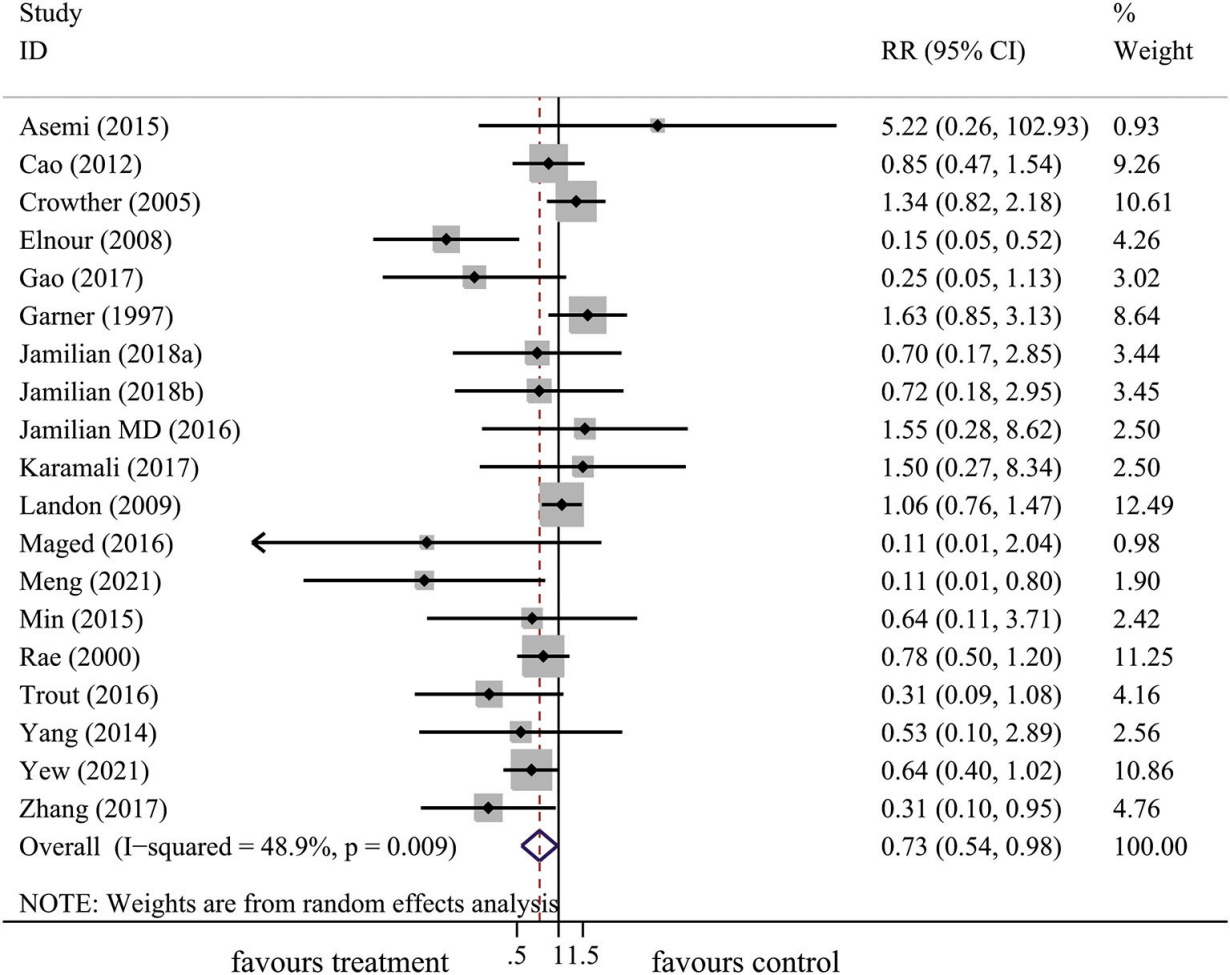
The forest plot demonstrated the effect of prenatal lifestyle intervention in women with GDM on the risk of neonatal hypoglycemia by pooling data from 18 studies.

In total, 18 articles included 19 trials that explored the effect of lifestyle intervention on the risk of neonatal hypoglycemia. With consideration of the heterogeneity, we used the random-effect model to get pooled estimates. Results from our meta-analysis suggested that lifestyle intervention during pregnancy could significantly reduce the risk of neonatal hypoglycemia (RR: 0.73, 95% CI: 0.54 to 0.98, *P* = 0.037) (Figure 4). The results of the *I*^*2*^ and chi^2^ test demonstrated that there was substantial heterogeneity in the primary meta-analysis (*I*^2^= 48.9%; *P* = 0.009) (Table 2). The Begg's and Egger's tests indicated no significant publication bias in the primary analysis (Table 2).

**Table 2:** Results of subgroup analysis and publication bias stratified by study characteristics.

**Variables**	**Trials (** ***n*** **)**	**RR (95% CI)**	***P** ^1^*	**Heterogeneity**		***P** ^3^*	
				***I**^2^* **(%)**	***P** ^2^*	**Begg's value**	**Egger's value**
Overall	19	0.73 (0.54 to 0.98)	0.037	48.9	0.009	0.529	0.713
**Age at pregnancy**							
≤ 30 years	8	0.52 (0.27 to 0.99)	0.046	51.4	0.044	1.000	0.159
> 30 years	11	0.81 (0.57 to 1.17)	0.272	52.3	0.021	0.161	0.537
**Gestational age at baseline**							
<14 weeks	3	0.30 (0.10 to 0.86)	0.025	0.00	0.614	1.000	0.730
14~28 weeks	7	0.66 (0.44 to 0.98)	0.039	0.7	0.419	0.548	0.242
≥ 28 weeks	6	0.85 (0.61 to 1.19)	0.337	50.6	0.072	0.260	0.178
**Fasting glucose level at baseline**							
<5.1 mmol/L	9	0.79 (0.57 to 1.07)	0.131	48.2	0.051	0.602	0.144
≥ 5.1 mmol/L	8	0.78 (0.42 to 1.46)	0.439	39.1	0.118	0.386	0.220
**Intervention type**							
Dietary intervention only	12	0.69 (0.48 to 0.98)	0.041	54.8	0.011	0.273	0.915
Dietary + other interventions	2	0.55 (0.04 to 6.83)	0.642	61.1	0.109	0.317	-
Dietary + exercise + other interventions	5	0.80 (0.41 to 1.55)	0.504	50.7	0.087	0.624	0.317

*P^1^ value for net change; P^2^ value for heterogeneity in the subgroup; P^3^ value for publication bias; significant p-values are highlighted in bold prints*.

### Subgroup and meta-regression analysis

**Figure 5:**
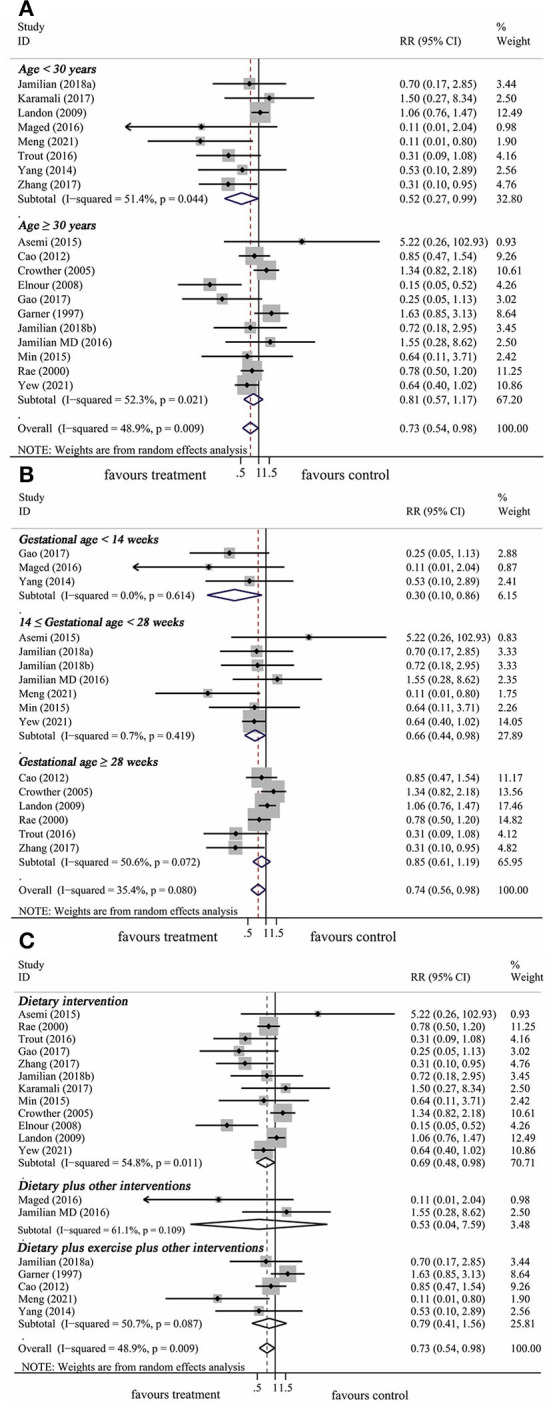
Subgroup analysis of prenatal lifestyle intervention on the risk of neonatal hypoglycemia stratified by maternal age at pregnancy **(A)**, gestational age at baseline **(B)**, and intervention types **(C)**.

The results of the subgroup analysis were summarized in Table 2. The results of subgroup analysis revealed that the effect of lifestyle intervention on the risk of neonatal hypoglycemia was influenced by maternal age at pregnancy and gestational age at trial entry. Lifestyle intervention was associated with a decrease in the risk of neonatal hypoglycemia only in studies with mothers younger than 30 years (RR: 0.52, 95% CI: 0.27–0.99, *P* = 0.046), but not in studies with mothers ≥ 30 years (RR: 0.81, 95% CI: 0.57–1.17, *P* = 0.272) (Figure 5A). Furthermore, significant reductions of risk of neonatal hypoglycemia post lifestyle intervention were observed only in studies with gestational age < 14 weeks (first trimester) at trial entry (RR: 0.30, 95% CI: 0.10–0.86, *P* = 0.025) and in studies with gestational age between 14 and 28 weeks (second trimester) at trial entry (RR: 0.66, 95% CI: 0.44–0.98, *P* = 0.039), but not in studies with gestational age ≥ 28 weeks (third trimester) at trial entry (RR: 0.85, 95% CI: 0.61–1.19, *P* = 0.337) (Figure 5B). No significant effect was observed in the subgroup results of baseline maternal fasting glucose level < 5.1 mmol/L or ≥ 5.1 mmol/L. In addition, results from subgrouping analysis by type of lifestyle intervention presented a reduction of neonatal hypoglycemia risk in studies using dietary intervention only (RR: 0.69, 95% CI: 0.48–0.98, *P* = 0.041), while this effect did not exist in dietary plus other interventions or dietary plus exercise plus other interventions (Figure 5C). The Begg's and Egger's tests indicated no significant publication bias in the above subgroup analyses (Table 2).

**Figure 6:**
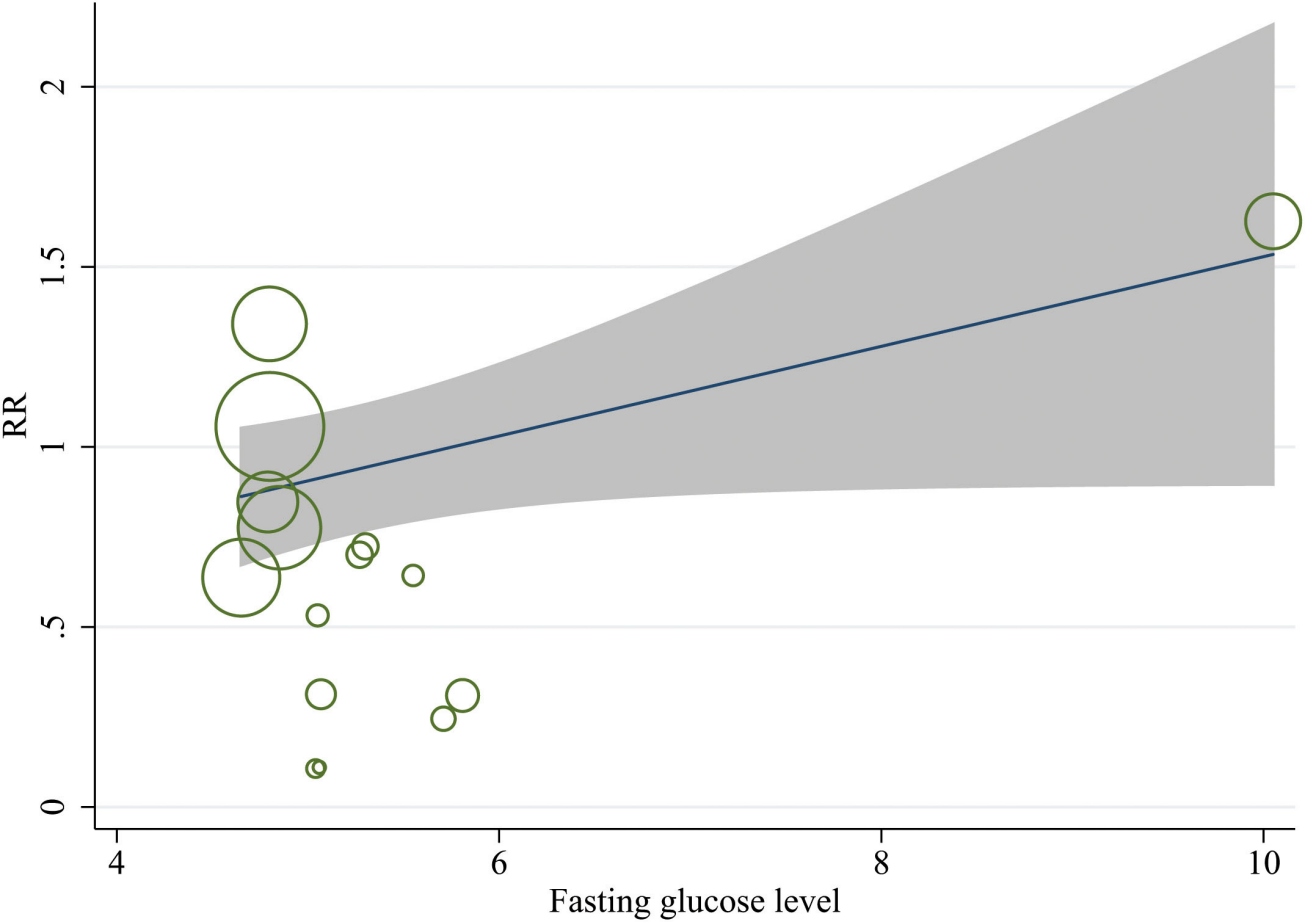
Meta-regression model for the effect of prenatal lifestyle intervention on the risk of neonatal hypoglycemia, adjusted for maternal fasting blood glucose levels at baseline.

A meta-regression analysis was conducted to explore the potential sources of heterogeneity. Among selected covariates, including maternal age at pregnancy, gestational age at trial entry, and maternal fasting glucose level at trial entry, the results of meta-regression analysis revealed that maternal fasting glucose level at trial entry was a potential confounder of the effect of lifestyle intervention on the risk of neonatal hypoglycemia, with adjusted R^2^ of 45.04% (Table 3 and Figure 6). There was no significant association between the risk ratio and other covariates listed in Table 3.

**Table 3:** Meta-regression analysis of potential moderators.

**Variables**	**Trials (** ***n*** **)**	**Coefficient**	**95% CI**	**P**	**Covariate adjusted R** ^2^
Age at pregnancy	19	−0.001	−0.239 to 0.237	0.994	-
Gestational weeks at baseline	16	0.051	−0.032 to 0.135	0.150	-
Fasting glucose level at baseline	15	0.128	−0.027 to 0.283	0.099	45.04%
Intervention type	19	0.062	−0.141 to 0.266	0.525	-

*Significant p-values are highlighted in bold prints*.

### Sensitivity analysis

**Figure 7:**
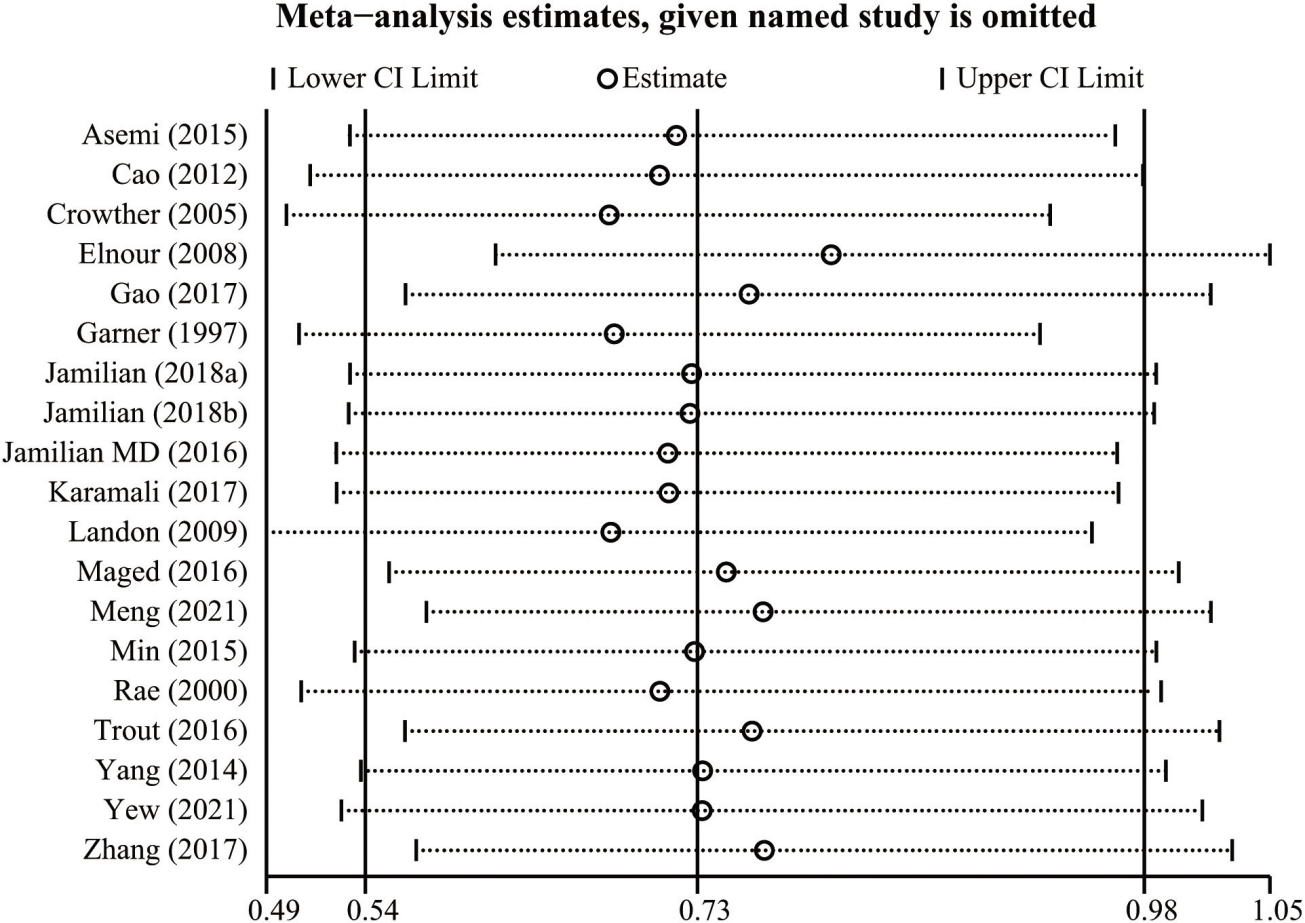
The result of leave-one-out cross-validation analysis.

Regarding the robustness of overall effect sizes, we performed a leave-one-out cross-validation for sensitivity analysis (Figure 7). The results of the leave-one-out cross-validation suggested that three studies (19, 20, 37) contributed most to the heterogeneity in the primary meta-analysis. After excluding these studies, the pooled results remained significant (RR: 0.71, 95% CI: 0.54–0.93, *P* = 0.012), thus the effect of the intervention on the risk of neonatal hypoglycemia might be underestimated due to heterogeneity between studies.

### Evidence level rated by GRADE

According to the GRADE protocol, as shown in Table 4, the evidence level of the overall meta-analysis was at a moderate level because significant heterogeneity existed.

**Table 4:** Grades of Recommendation, Assessment, Development and Evaluation (GRADE) quality of evidence.

**Outcome**	**Risk** ** of** **Bias**	**Inconsistency**	**Indirectness**	**Imprecision**	**Publication** **bias**	**Effect** **Size**	**Plausible** **residual confounding**	**Dose-response** **gradient**	**GRADE** **rating**
	**None**	**serious ^a^**	**None**	**None**	**none**	**none**	**none**	**None**	**Moderate**

*Significant and unexplained variability exists in the primary meta-analysis*.

## Discussion

All in all, there was “moderate” quality evidence from 18 RCTs indicating that prenatal lifestyle intervention in women with GDM was associated with a 27% decreased risk of having a baby with hypoglycemia. Subgroup analysis further demonstrated that the reduced risk of neonatal hypoglycemia post lifestyle intervention was observed only in studies with subjects younger than 30 years, initiated in the first or second trimester, and with dietary intervention. In addition, meta-regression analysis revealed that maternal fasting glucose levels at trial entry were positively associated with the risk ratio of neonatal hypoglycemia post lifestyle intervention.

To the best of our knowledge, this is the first comprehensive systematic review and meta-analysis that evaluated the effect of prenatal lifestyle intervention in women with GDM on the risk of neonatal hypoglycemia. Unlike our results, relevant systematic reviews to date did not find any significant benefit of lifestyle intervention for neonatal hypoglycemia. The meta-analysis from Cochrane (55) included six studies, that reported no significant association between the lifestyle treatment of GDM and the risk of neonatal hypoglycemia. Five of the six studies were also included in our meta-analysis, but the remaining one study (51) was excluded because the study population was women with impaired glucose tolerance rather than patients with GDM. In addition, a systematic review (56) reported no difference between infants exposed to control or diet and exercise interventions before birth for the risk of hypoglycemia, which was based on two studies with overweight or obese women. Unlike our findings, another meta-analysis (57) based on two studies, which were both included in our review, found that dietary intervention in patients with GDM did not change the neonatal outcome of hypoglycemia. In our present study, we found that only dietary intervention in patients with GDM could reduce the risk of neonatal hypoglycemia, but not dietary intervention plus other interventions. This might be due to dietary advice or counseling (rather than supplements) being the main form of dietary intervention in the subgroups of dietary plus other interventions with or without exercise (one (19) of two (19, 26) studies in the group of dietary plus other interventions and three (20, 24, 43) of five (20, 21, 23, 24, 43) studies in the group of dietary plus exercise plus other interventions applied individualized dietary advice or counseling intervention). Since the small number of studies with other interventions included in our study, future large-scale RCTs are still required to further explore the best pattern of lifestyle intervention during pregnancy to reduce the risk of neonatal hypoglycemia. There is no relevant report describing long-term follow-up of hypoglycemia outcomes after changing lifestyle during pregnancy.

The role of lifestyle intervention has been greatly appreciated as a clinical treatment in GDM (58). In our present meta-analysis, prenatal lifestyle intervention resulted in a significant reduction in the risk of neonatal hypoglycemia. Achieving glycemic control in women with GDM is critical for reducing the risk of neonatal hypoglycemia (59). Notably, the glucose threshold recommended by recent guidelines (1, 60, 61) is lower than previously recommended (62, 63) for the diagnosis of GDM. Therefore, less severe hyperglycemia has been classified as GDM in recent years, which is also conducive to the effect of lifestyle intervention. Maternal age is known to affect the outcomes of pregnancies (64). Recently, a meta-analysis of 24 studies showed that the risk of GDM increased by 7.90% with each-one year increase in maternal age from 18 (65). The increase in maternal age is also related to the incidence of macrosomia (66), small for gestational age (67), and cesarean section (68), which are all independent risk factors for neonatal hypoglycemia (69). Consequently, we postulated that the adverse outcomes associated with advanced maternal age might lead to an increased incidence of neonatal hypoglycemia, thereby obscuring the effect of lifestyle interventions. Our meta-analysis could suggest that pregnant women younger than 30 years might be an appropriate population to observe the improved effect on neonatal hypoglycemia post lifestyle intervention.

Traditionally, screening tests for the diagnosis of GDM are performed at 24–28 weeks of gestation. A prospective cohort study of 4,069 women showed that the increase in fetal growth was not obvious when GDM was diagnosed at 20 weeks of gestation, but it was significantly increased when GDM was diagnosed ≥ 28 weeks (70). In addition, fetal growth in obese women increased when GDM was diagnosed at 20 weeks of gestation (70). This indicated that late diagnosis might miss the opportunity for intervention, especially for a high-risk population. Our findings suggest that lifestyle intervention might be effective for reducing the risk of neonatal hypoglycemia when initiated before 28 weeks of gestation. And this increases the question of the current diagnosis time of GDM. It is necessary to conduct GDM screening in early pregnancy to increase the opportunity of benefiting from early intervention. Therefore, we suggest that the diagnostic criteria of GDM in early pregnancy should be determined reasonably.

An observational epidemiological international multi-ethnic investigation found that intrauterine exposure to higher levels of glucose was associated with childhood obesity and insulin resistance, which was independent of maternal BMI and family history of diabetes (71, 72). A prospective study of patients with GDM found that infants whose mothers had the lowest blood glucose levels before and at birth had the lowest incidence of neonatal hypoglycemia (73). Likewise, our results of meta-regression analysis suggested that the risk of neonatal hypoglycemia post lifestyle intervention was lower in patients with GDM having lower maternal fasting glucose levels at trial entry. Thus, maternal fasting glucose levels at trial entry might be the potential source of heterogeneity among studies.

Our meta-analysis has some limitations. One possible limitation may be related to the different GDM diagnostic criteria used in the included studies, which might lead to the inevitable existence of heterogeneity between studies. In fact, the diagnostic criteria of GDM have been controversial (60). In addition, there were a variety of lifestyle interventions included in this study, and the number of studies reporting each intervention was limited, so we cannot determine which specific intervention is more effective. Furthermore, we could not find out how much the degree of hypoglycemia is worse or improves with a change in lifestyle due to the limitation of data. Lastly, significant heterogeneity among studies was found. According to the result of the meta-regression, the heterogeneity might be attributed to the differences in maternal fasting glucose levels at trial entry.

## Conclusion

This current meta-analysis of 18 RCTs demonstrated that lifestyle intervention during pregnancy in women with GDM significantly reduced the risk of neonatal hypoglycemia, especially when subjects were younger than 30 years old, or lifestyle intervention initiated before the third trimester, or with dietary intervention. However, future well-designed large-scale trials are still required to further explore the best pattern of lifestyle intervention and to determine the proper early diagnostic criteria for GDM.

## Data availability statement

The original contributions presented in the study are included in the article/supplementary material, further inquiries can be directed to the corresponding authors.

## Author contributions

Y-HW, H-HZ, ZZ, and ZY designed and conducted the research, collected and analyzed the data, and wrote the article. Y-HW, H-HZ, ZZ, ZY ZN, JT, ZY, and SZ helped with the data interpretation, contributed to the discussion, and revised the article. ZZ and ZY had primary responsibility for the final content of the manuscript. All authors participated in critically revising and approving the final manuscript.

## Conflict of interest

The authors declare that the research was conducted in the absence of any commercial or financial relationships that could be construed as a potential conflict of interest. The handling editor LQ declared a shared parent affiliation with the author ZZ at the time of the review.

## Publisher's note

All claims expressed in this article are solely those of the authors and do not necessarily represent those of their affiliated organizations, or those of the publisher, the editors and the reviewers. Any product that may be evaluated in this article, or claim that may be made by its manufacturer, is not guaranteed or endorsed by the publisher.
