# Supplementary material for: Lifestyle intervention during pregnancy in patients with gestational diabetes mellitus and the risk of neonatal hypoglycemia: A systematic review and meta-analysis
Source: Front Nutr. 2022 Jul 28;9:962151. doi: 10.3389/fnut.2022.962151 (PMC9376328; doi:10.3389/fnut.2022.962151)
Supplement: Supplementary file 1 [file Table_1.DOCX]

**Supplementary table 1** Detailed search strategies.

Example search strategy for the PubMed database.

**((Diet[Mesh]) OR (Diets[Title/Abstract]) OR (Diet Therapy[Mesh]) OR (Diet Therapies[Title/Abstract]) OR (Therapy, Diet[Title/Abstract]) OR (Diet Therapy, Restrictive[Title/Abstract]) OR (Restrictive Diet Therapies[Title/Abstract]) OR (Therapy, Restrictive Diet[Title/Abstract]) OR (Restrictive Diet Therapy[Title/Abstract]) OR (Restriction Diet Therapies[Title/Abstract]) OR (Diet Therapies, Restriction[Title/Abstract]) OR (Diet Therapy, Restriction[Title/Abstract]) OR (Therapy, Restriction Diet[Title/Abstract]) OR (Restriction Diet Therapy[Title/Abstract]) OR (Dietary Restriction[Title/Abstract]) OR (Dietary Restrictions[Title/Abstract]) OR (Restriction, Dietary[Title/Abstract]) OR (Dietary Modification[Title/Abstract]) OR (Dietary Modifications[Title/Abstract]) OR (Modification, Dietary[Title/Abstract]) OR (Diet Modification[Title/Abstract]) OR (Diet Modifications[Title/Abstract]) OR (Modification, Diet[Title/Abstract]) OR (Nutrition[Title/Abstract]) OR (Nutrition Therapy[Mesh]) OR (Therapy, Nutrition[Title/Abstract]) OR (Medical Nutrition Therapy[Title/Abstract]) OR (Nutrition Therapy, Medical[Title/Abstract]) OR (Therapy, Medical Nutrition[Title/Abstract]) OR (Lifestyle[Mesh]) OR (Life Styles[Title/Abstract]) OR (Lifestyle[Title/Abstract]) OR (Lifestyles[Title/Abstract]) OR (Life Style Induced Illness[Title/Abstract]) OR (Lifestyle Factors[Title/Abstract]) OR (Factor, Lifestyle[Title/Abstract]) OR (Lifestyle Factor[Title/Abstract])) OR ((Exercise[Mesh]) OR (Exercise, Physical[Title/Abstract]) OR (Exercises, Physical[Title/Abstract]) OR (Physical Exercise[Title/Abstract]) OR (Physical Exercises[Title/Abstract]) OR (Physical Activity[Title/Abstract]) OR (Exercise, Isometric[Title/Abstract]) OR (Exercises, Isometric[Title/Abstract]) OR (Isometric Exercises[Title/Abstract]) OR (Isometric Exercise[Title/Abstract]) OR (Exercise, Aerobic[Title/Abstract]) OR (Aerobic Exercises[Title/Abstract]) OR (Exercises, Aerobic[Title/Abstract]) OR (Aerobic Exercise[Title/Abstract]) OR (Exercise Therapy[Mesh]) OR (Therapy, Exercise[Title/Abstract]) OR (Exercise Therapies[Title/Abstract]) OR (Therapies, Exercise[Title/Abstract]) OR (Exercise Movement Techniques[Mesh]) OR (Movement Techniques, Exercise[Title/Abstract]) OR (Exercise Movement Technics[Title/Abstract]) OR (Pilates-Based Exercises[Title/Abstract]) OR (Exercises, Pilates-Based[Title/Abstract]) OR (Pilates Based Exercises[Title/Abstract]) OR (Pilates Training[Title/Abstract]) OR (Sports[Mesh]) OR (Athletics[Title/Abstract]) OR (Athletic[Title/Abstract]) OR (Physical Fitness[Mesh]) OR (Fitness, Physical[Title/Abstract]) OR (Motor Activity[Mesh]) OR (Activities, Motor[Title/Abstract]) OR (Motor Activities[Title/Abstract])) AND ((Pregnancy[Mesh]) OR (Pregnant[Title/Abstract]) OR (Pregnancies[Title/Abstract]) OR (Gravid[Title/Abstract]) OR (Gestation[Title/Abstract]) OR (Pregnant Women[Mesh]) OR (Woman, Pregnant[Title/Abstract]) OR (Women, Pregnant[Title/Abstract]) OR (Mothers[Mesh]) OR (Mothers' Clubs[Title/Abstract]) OR (Maternal Exposure[Mesh]) OR (Exposure, Maternal[Title/Abstract]) OR (Maternal Exposures[Title/Abstract]) OR (Prenatal Exposure Delayed Effects[Mesh]) OR (Delayed Effects, Prenatal Exposure[Title/Abstract]) OR (Late Effects, Prenatal Exposure[Title/Abstract])) AND ((Diabetes Mellitus[Mesh]) OR (Diabetes[Title/Abstract]) OR (DM[Title/Abstract]) OR (Glucose[Mesh]) OR (Anhydrous Dextrose[Title/Abstract]) OR (Insulin[Mesh]) OR (Hyperglycemia[Mesh]) OR (Hyperglycemia, Postprandial[Title/Abstract]) OR (Congenital Hyperinsulinism[Title/Abstract])) AND ((hypoglycemia[Title/Abstract]) OR (Neonatal hypoglycemia[Title/Abstract]) OR (Offspring[Title/Abstract]) OR (offspring outcomes[Title/Abstract]) OR (maternal outcomes[Title/Abstract]) OR (Newborn outcomes[Title/Abstract]) OR (Child[Mesh]) OR (Children[Title/Abstract]) OR (Infant, Newborn[Title/Abstract]) OR (Infants, Newborn[Title/Abstract]) OR (Newborn Infant[Title/Abstract]) OR (Newborn Infants[Title/Abstract]) OR (Newborns[Title/Abstract]) OR (Newborn[Title/Abstract]) OR (Neonate[Title/Abstract]) OR (Neonates[Title/Abstract])) AND ((randomized controlled trial [Publication Type])OR (randomized) OR (placebo))**

Example search strategy for other databases.

**((Diet OR Diets OR ”Diet Therapy” OR ”Diet Therapies” OR ”Therapy, Diet” OR ”Diet Therapy, Restrictive” OR “Restrictive Diet Therapies” OR “Therapy, Restrictive Diet” OR “Restrictive Diet Therapy” OR “Restriction Diet Therapies” OR “Diet Therapies, Restriction” OR “Diet Therapy, Restriction” OR “Therapy, Restriction Diet” OR “Restriction Diet Therapy” OR “Dietary Restriction” OR “Dietary Restrictions” OR “Restriction, Dietary” OR “Dietary Modification” OR “Dietary Modifications” OR “Modification, Dietary” OR “Diet Modification” OR “Diet Modifications” OR “Modification, Diet” OR “Nutrition” OR “Nutrition Therapy” OR “Therapy, Nutrition” OR “Medical Nutrition Therapy” OR “Nutrition Therapy, Medical” OR “Therapy, Medical Nutrition” OR “Lifestyle” OR “Life Styles” OR “Lifestyle” OR “Lifestyles” OR “Life Style Induced Illness” OR “Lifestyle Factors” OR “Factor, Lifestyle” OR “Lifestyle Factor”) OR (Exercise OR Exercises OR “Exercise, Physical” OR “Exercises, Physical” OR “Physical Exercise” OR “Physical Exercises” OR “Physical Activity” OR “Exercise, Isometric” OR “Exercises, Isometric” OR “Isometric Exercises” OR “Isometric Exercise” OR “Exercise, Aerobic” OR “Aerobic Exercises” OR “Exercises, Aerobic” OR “Aerobic Exercise” OR “Exercise Therapy” OR “Therapy, Exercise” OR “Exercise Therapies” OR “Therapies, Exercise” OR “Exercise Movement Techniques” OR “Movement Techniques, Exercise” OR “Exercise Movement Technics” OR “Pilates-Based Exercises” OR “Exercises, Pilates-Based” OR “Pilates Based Exercises” OR “Pilates Training” OR “Training, Pilates” OR Sports OR Sport OR Athletics OR Athletic OR “Physical Fitness” OR “Fitness, Physical” OR “Motor Activity” OR “Activities, Motor” OR “Activity, Motor” OR “Motor Activities”)) AND ((Pregnancy OR Pregnant OR Pregnancies OR Gravid OR Gestation OR “Pregnant Women” OR “Pregnant Woman” OR “Woman, Pregnant” OR “Women, Pregnant” OR Mothers OR Mother OR “Maternal Exposure” OR “Exposure, Maternal” OR “Exposures, Maternal” OR “Maternal Exposures” OR “Prenatal Exposure Delayed Effects” OR “Delayed Effects, Prenatal Exposure” OR “Late Effects, Prenatal Exposure”) AND (“Diabetes Mellitus” OR “Diabetes” OR “DM” OR “Glucose” OR “Anhydrous Dextrose” OR “Insulin” OR “Hyperglycemia” OR “Hyperglycemia, Postprandial” OR “Congenital Hyperinsulinism”)) AND (“Offspring” OR “offspring outcomes” OR “maternal outcomes” OR “Newborn outcomes” OR “Child” OR “Children” OR “Infant, Newborn” OR “Infants, Newborn” OR “Newborn Infant” OR “Newborn Infants” OR “Newborns” OR “Newborn” OR “Neonate” OR “Neonates” OR “hypoglycemia” OR “Neonatal hypoglycemia”) AND ("randomized controlled trial" OR "randomized" OR "placebo")**
